# Academic stress among female university students in China: a qualitative study of coping through psychological capital and social support

**DOI:** 10.1186/s40359-026-05482-6

**Published:** 2026-10-05

**Authors:** Irum Zeb, Song Ye, Aashiq Khan

**Affiliations:** 1 School of Political Science and Public Administration, Henan Normal University, Xinxiang, China; 2 School of Education, Henan Normal University, Xinxiang, China; 3 School of Political Science and Public Administration, Henan Normal University, Xinxiang, China

**Keywords:** Academic stress, PsyCap, Social support, Female students, Perceptions

## Abstract

**Background:**

Academic stress is a pervasive psychological concern among university students, and female students often face additional stressors related to societal expectations, academic demands, and gender roles. However, there is limited qualitative research exploring how female students understand and cope with academic stress in Chinese higher education. This study aimed to explore female university students lived experiences of academic stress and the role of psychological capital and social support in coping.

**Methods:**

A qualitative study was conducted through semi-structured interviews with 15 female students at a Chinese university. Interviews were conducted in both Chinese and English. Interviews were audio-recorded, transcribed verbatim, and analyzed using thematic analysis guided by Braun and Clarke’s six-phase framework to identify patterns and themes related to academic stress and coping strategies. The analysis was primarily inductive but theoretically informed by the study’s focus on psychological capital, social support, and gendered experiences of academic stress.

**Results:**

Participants reported that academic stress was driven by high academic expectations, family responsibilities, and sociocultural gender norms. Psychological capital, including resilience, self-efficacy, optimism, and hope, helped students reframe stress and maintain motivation. Social support from family, peers, and mentors provided emotional and practical assistance, buffering stress and promoting adaptive coping. Across participants’ accounts, both psychological capital and social support were described as resources for coping with academic demands. Across participants’ accounts, both psychological capital and social support were described as resources for coping with academic demands.

**Conclusions:**

Female university students experience academic stress shaped by academic demands and gendered family and social expectations. Psychological capital and social support were described as important coping resources. These findings suggest potential implications for university support strategies that are responsive to female students’ academic and gender-related pressures.

## Introduction

Academic stress is a significant global issue affecting university students’ well-being and academic success. The pressures of meeting academic demands, managing personal responsibilities, and maintaining social relationships contribute to students’ stress [1]. Academic stress among university students generally encompasses demands related to coursework, exams, academic performance, and the balance between extracurricular and personal responsibilities [2]. However, for female students, these stressors are often compounded by societal and familial expectations that may place additional pressure on them [3]. Gender specific stressors in Chinese universities include cultural expectations around femininity, caregiving roles, and the pressure to maintain traditional family structures while excelling academically [4]. Female students often face the burden of meeting high academic standards while adhering to gendered norms of modesty and family responsibilities [4]. Additionally, the underrepresentation of women in academic leadership roles in Chinese universities can reinforce gender biases, contributing to feelings of marginalization and exclusion from the decision-making process [5]. These gendered challenges significantly affect female students’ ability to manage academic stress, creating a need for research that explores how they navigate these complex stressors and the coping strategies they employ [6].

Psychological capital (resilience, hope, self-efficacy, and optimism) is a critical resource for managing academic stress [7]. Resilience enables female students in Chinese universities to recover from setbacks despite societal challenges [8]. However, gendered expectations, like family responsibilities, can influence how resilience is expressed. Hope and optimism help female students persist in their studies, despite barriers [9]. Self-efficacy and belief in one’s abilities to succeed are important for coping with academic stress [10]. In gender biased environments, female students experience lower self-efficacy, affecting their stress management strategies [11].

In addition to PsyCap, social support is a vital resource for managing academic stress [12]. Social support, including emotional, informational, and practical help from family, friends, and peers, can reduce the negative impact of stress on female students [13]. Female students, particularly in cultures with gendered expectations, social support networks play an important role in managing academic stress. Family support is vital for female students, offering emotional and practical help [14]. However in patriarchal societies such as China, family expectations often conflict with academic goals prioritizing marriage over education [15]. Thus, family support can both alleviate and contribute to stress. Peer support also helps to reduce academic stress by providing emotional support and social connections, especially in a collectivist culture like China [14].

Our study adopts a qualitative research approach, which allows an in-depth exploration of how female students understand and experience academic stress within their social and academic contexts. This approach is appropriate for examining how gender roles, family expectations, and institutional experiences are reflected in participants’ accounts. Given these gendered academic pressures, it is important to understand how female university students experience academic stress and how internal psychological resources and external support systems help them cope in everyday academic life. Accordingly, this qualitative study addresses the following research questions:

RQ1: How do female students experience and make sense of academic stress?

RQ2: How does psychological capital support their coping with academic stress?

RQ3: How does social support help them cope, and how do gendered barriers shape these coping processes?

### Literature review

Academic stress is a significant concern in higher education, particularly for female students who experience challenges due to the intersection of personal, academic, and societal stressors [16]. While academic stress is commonly influenced by academic demands, workload, and performance expectations, gendered expectations compound the stress experienced by female students [17]. Female students often face the dual burden of meeting academic expectations while conforming to traditional gender norms, requiring them to balance familial and academic roles. This gender dimension of academic stress has been explored in several studies [18]. In the context of Chinese universities, females are particularly affected by these compounded stressors as they must navigate both academic pressures and societal expectations regarding their familial responsibilities [19].

Psychological capital, which includes resilience, hope, self-efficacy, and optimism, has been recognized as an essential factor in managing stress among university students [20]. Previous studies have suggested that these psychological resources are particularly valuable for female students navigating academic stress, as they help them maintain motivation and persevere through setbacks [21]. However, gendered expectations in Chinese universities impact the way these psychological resources are used, with female students potentially facing additional barriers in building and utilizing their psychological capital [20]. Furthermore, social support is another key factor in alleviating academic stress, as it provides students with emotional, informational, and instrumental support [22]. For female students, social support from family, peers, and mentors is particularly important in mitigating the stress associated with gendered expectations and academic challenges [23]. However, social support for female students can be a double-edged sword in societies with strong patriarchal values. Familial support may sometimes reinforce traditional gender roles, with families placing pressure on female students to prioritize family responsibilities over academic success [24]. The academic environment plays a pivotal role in shaping how female students cope with academic stress [25]. In Chinese universities, the lack of female representation in academic leadership roles perpetuates gender biases and limits the support available for female students [26]. Female students often feel marginalized in a male-dominated academic field, which can exacerbate feelings of isolation and stress [27]. Moreover, the rigid expectations surrounding gender roles in educational settings often lead to additional stressors such as moral policing and limited opportunities for social engagement. These gendered barriers, coupled with academic pressures, create a unique set of challenges for female students, which is often underexplored in the traditional model of academic stress.

This study examines the lived experiences of female students in Chinese universities, focusing on the gendered nature of academic stress and how female students navigate these challenges. This approach allows for the identification of structural and cultural barriers that limit women’s opportunities in academia and the exploration of resources such as psychological capital and social support that help female students cope with stress. By incorporating the voices of female students, qualitative research provides rich insights into how gendered experiences influence their academic success and well-being.

## Methods

The sample consisted of 15 female students enrolled at a Chinese university, with recruitment ending after 15 interviews when no substantively new codes or themes were identified, consistent with the study’s saturation criterion [28]. All participants identified as female (*n* = 15; 100%) and were aged between 18 and 26 years. Participants were purposively recruited from three related social science disciplines: Education, Psychology, and Sociology. These disciplines were selected because of their relevance to the study’s focus on academic stress and gendered experiences. All participants were recruited from the same university. The disciplinary concentration of the sample should therefore be considered when interpreting the transferability of the findings. Five students were recruited from each discipline. All participants were full-time students who self-reported experiencing moderate to high levels of academic stress at the time of data collection. Demographic information for the full sample is presented in Table 1.

**Table 1:** Demographic Characteristics of Interview Participants (*n* = 15)

Variable	Category	*n*	%
Gender	Female	15	100
Age Group	18–21 years	5	33.3
	22–25 years	8	53.3
	26 years and above	2	13.3
Discipline	Education	5	33.3
	Psychology	5	33.3
	Sociology	5	33.3
Study level	Undergraduate	7	46.7
	Master	4	26.7
	PhD	4	26.7

### Procedure

Participants were recruited from a Chinese university. Purposive sampling was used to recruit eligible female students from Education, Psychology, and Sociology. Potential participants were approached directly by the researcher and invited to participate. The researcher had no prior relationship with the participants before recruitment. Eligibility criteria included: (a) current full-time enrollment at the university, and (b) self-identification as female. Participants received study information and provided written informed consent before the interview. Individual semi-structured interviews were then scheduled at a convenient time and conducted in a quiet campus room. Each interview lasted approximately 40–50 min and was audio-recorded with the participant’s permission. The recordings were transcribed verbatim for analysis, and study data were securely stored to protect participant confidentiality.

### Interview protocol

All interviews were conducted face-to-face in a quiet and comfortable room to ensure privacy and allow participants to speak openly. The first author led the interviews. The first author (AK) is a male international researcher and had no prior relationship with the participants before recruitment or data collection. This was considered relevant because the interviews addressed potentially sensitive experiences of gender and academic stress. Before beginning, participants were reminded that their participation was voluntary, that they could skip any question, and that they could withdraw from the interview at any time without consequence, with participants’ consent.

Interviews were conducted in both Chinese and English. Chinese-language interview material was translated into English by the second author (SY), a female Chinese professor. Translation accuracy was assessed through author review of the translated material.

The interview began with introductory questions about the participants’ backgrounds, including their field of study and year level. Following this, participants were asked to describe their experiences as female students and to share specific situations in which they had felt academic stress. They were then guided to reflect on how they typically respond to academic challenges, including their use of coping strategies and elements of psychological capital such as resilience, hope, optimism, and self-efficacy. The next section of the interview explored the role of social support in managing academic stress. Participants were asked about the support they received from family, friends, peers, and mentors, as well as their perceptions of university mental health services. The interview then shifted to gendered experiences of academic stress, asking participants whether being a female student influenced how they experienced and coped with academic pressure, and whether they had encountered gender-related expectations or challenges during their studies.

### Ethical considerations

Participants received information about the study’s purpose, procedures, and intended use of the data before providing written informed consent. Participation was voluntary, and participants were informed that they could skip any question or withdraw from the study without consequence. Interviews were conducted in a private setting, and participant confidentiality was protected through the use of participant codes or pseudonyms in transcripts and reporting. Study data were securely stored and handled confidentially. Because the interviews addressed potentially sensitive experiences of academic stress and gender-related pressures, participants were informed that the interview could be paused or stopped if they experienced distress. If a participant became uncomfortable or distressed, the interviewer was prepared to pause or terminate the interview according to the participant’s preference. No formal referral procedure or study-specific referral pathway to psychological support services was available as part of the research protocol. These procedures concerned the ethical conduct and protection of participants and were separate from the methodological procedures used to assess and strengthen the credibility of the qualitative analysis.

### Researcher positionality and reflexivity

The research team occupied both insider and outsider positions in relation to the study context. The first author is a male international postdoctoral researcher, the second author is a female Chinese professor and Dean in the School of Education, and the third author is also a female international postdoctoral researcher. The second author therefore brought cultural, linguistic, and institutional knowledge of Chinese higher education, while the first and third authors contributed international and cultural outsider perspectives. Because the first author, a male researcher, conducted the interviews with female students about potentially sensitive gendered experiences, his gender and international status have influenced how participants described/disclosed some experiences. This possibility was considered during interpretation of the interview data. Conversely, the involvement of two female researchers, including a Chinese female institutional insider, provided additional perspectives when interpreting participants’ accounts of gender-related expectations and academic stress. The different cultural positions of the research team also created the possibility that culturally specific meanings could be interpreted through different assumptions. During analysis and theme development, interpretations were discussed across the research team and compared with the interview data. The second author’s Chinese linguistic and cultural knowledge, including her role in translating Chinese-language interview material, supported the interpretation of culturally specific meanings, while the international researchers contributed alternative perspectives. These different gender, cultural, linguistic, and institutional positions were considered during the review and refinement of themes to reduce the influence of any single researcher’s perspective on the interpretation of participants’ accounts.

### Analytical approach

The data were analyzed using thematic analysis, which is effective for identifying and analyzing patterns in qualitative data [29]. This method was selected for its flexibility and rigor in analyzing participants’ experiences, particularly their coping strategies and psychological resources related to academic stress. The study examines how gendered experiences influence stress and coping mechanisms. The analysis was conducted as a team-based thematic analysis guided by Braun and Clarke’s six-phase framework [29], rather than as reflexive thematic analysis. The coding was primarily inductive, allowing codes and patterns to be developed from participants’ accounts. However, the analysis was also theoretically informed because psychological capital, social support, and gendered experiences of academic stress were explicitly addressed in the research questions and interview guide. These concepts therefore served as sensitizing areas of inquiry rather than fixed predetermined themes, and the coding remained open to experiences and patterns beyond these areas.

The analysis followed a six-phase process [30]. In Phase 1, authors immersed themselves in the data by reviewing interviews, noting initial insights, and identifying emerging patterns. Phase 2 involved open coding, during which 60 initial codes were generated from participants’ interview data. These codes were derived inductively, capturing key ideas and concepts related to stress, coping strategies, and psychological resources. A working codebook was developed from these initial codes and refined during the analysis. Coding and codebook development were undertaken by the research team, with coding decisions reviewed through team discussion. In phase 3, patterns across the codes were identified, with team discussions used to review coding decisions, refine the working codebook, and develop candidate themes. Codes were grouped into candidate themes according to shared patterns of meaning rather than simply reproducing the sections of the interview guide. Codes concerning the experience and consequences of academic stress were grouped under Theme 1; codes describing internal psychological coping resources were grouped under Theme 2; codes concerning relational and interpersonal sources of support formed Theme 3; and codes specifically reflecting gendered family roles, emotional demands, and gender bias formed Theme 4. During theme refinement, the boundaries between Themes 1 and 4 were reviewed so that general experiences and consequences of academic stress were distinguished from the specifically gendered conditions that shaped stress and coping. Saturation was considered achieved after 15 interviews, when no new codes or themes emerged in subsequent transcripts, suggesting that further interviews would not yield additional insights. The remaining data exhibited similar patterns, confirming that the sample size was sufficient for a comprehensive understanding of the topic. In Phase 4, the themes were refined through an iterative process, with comparisons made between raw data and the developing themes, followed by team review to ensure that the themes were adequately supported by participants’ accounts. During Phases 5 and 6, the themes were finalized with clear definitions through team consensus and review. Member checking was conducted by inviting a subset of participants to review the transcribed data and the emerging themes. Participant feedback was used as an additional credibility check on the interpretation of their experiences. Member checking was treated as a methodological credibility procedure and was used to consider whether the developing interpretations adequately reflected participants’ accounts.

## Results

The following section presents the study’s findings, exploring the role of psychological capital and social support in coping with academic stress among female students at Chinese universities. The findings are categorized into four main themes, with corresponding subthemes that provide deeper insight into the participants’ experiences. The themes represent related but analytically distinct patterns: Theme 1 describes how academic stress was experienced, Theme 2 explains the psychological resources used in coping, Theme 3 identifies how social support facilitated coping, and Theme 4 examines how gendered barriers shaped these coping processes. Participants’ abbreviations were used throughout the findings section to protect confidentiality and de-identify the reported quotations: FS for female students. Quotations originally provided in Chinese are presented in English translation by the second author, whereas quotations provided in English are presented in their original interview language. Table 2 shows the main themes and sub-themes that emerged from the data.

**Table 2:** Themes and subthemes identified in the study

Theme	Theme Definition	Example
1. Experiences of Academic Stress	Academic stress was experienced as persistent pressure associated with academic performance and multiple demands and was reflected in fatigue, anxiety, difficulty concentrating, and sleep problems.	*“There is always this feeling that I cannot afford to fail… I can never relax. My family expects me to help at home.”*
2. Psychological Capital and Coping Strategies	Female students drew on internal psychological resources- resilience, persistence, optimism, hope, and self-efficacy to push through difficult periods, reframe stress as temporary, and maintain motivation toward long-term goals.	*“When stress becomes overwhelming*,* I remind myself that I have managed challenging situations before and I can do it again… it’s about finding a way to keep moving forward.”*
3. Role of Social Support	Emotional and practical support from family, friends, peers, and mentors acted as a buffer against academic stress by offering reassurance, shared study, guidance, and a sense of not facing university pressures alone.	*“Whenever I feel overwhelmed*,* my family reminds me to stay calm… my friends study with me*,* and that makes the workload feel lighter.”*
4. Gendered Barriers Shaping Coping with Academic Stress	Gendered norms shaped not only participants’ exposure to academic stress but also their ability to cope with it. Caregiving responsibilities, emotional demands, and perceived gender bias consumed time and emotional resources and required additional effort alongside academic work.	*“At home*,* I have to look after my younger siblings and do the chores*,* and at the university*,* I am expected to perform at the highest level. It feels like I am pulled in two directions at once.”*

### Experiences of academic stress

#### Awareness of academic stress

Awareness of academic stress in female students involves recognizing both the academic and social pressures they face during their university experience. Students viewed academic stress as something inherent in the higher education (HE) system, compounded by gendered expectations. Female students described academic stress not just as pressure from assignments and exams but as a multifaceted experience involving societal and familial pressures, such as the expectation to succeed academically while fulfilling traditional domestic roles.

In response to questions about their awareness of academic stress, most participants mentioned that external responsibilities, particularly those related to gendered expectations, exacerbate academic pressure, as FS3 voiced:

*"There is always this feeling that I can not afford to fail. Each assignment feels like adefining moment, and with the exam coming up. I can never relax. My family expectsme to help at home."*

This suggests that these gendered expectations not only increase academic stress but alsohinder the ability to relax and recharge, as students feel obligated to balance their academiccommitments with familial roles. As FS7 stated.

*"Society expects us to handle everything, and that only adds to the stress. It feels likethere is always something to prove, even when we are exhausted.” FS7 describedacademic stress as not only related to schoolwork but also to the pressure to fulfillmultiple roles, such as being a perfect student, daughter, and sister.*

This reflects the added burden of social expectations on female students, where academic stressis compounded by the need to meet family and social standards. The findings suggest that theconstant pressure to excel in various roles increases overall stress and contributes to feelings ofexhaustion, reinforcing the challenge of balancing multiple responsibilities.

#### Gender academic stress

The gendered aspect of academic stress emerged as a significant theme throughout the interview. A female student reported experiencing additional pressures not typically faced by male students. These pressures included managing household responsibilities, fulfilling family roles, and navigating societal expectations about gender roles. Many of the female students noted how their academic journeys were complicated by their responsibilities at home, where societal norms often dictate that females take on primary caregiving roles, leading to a double burden, as FS9 mentioned.

*“At home*,* I have to look after my younger siblings*,* do the chores*,* and help my parents*,* and at the university*,* I am expected to perform at the highest level. It feels like I am pulled in two directions at once and there is no time to focus on myself and my study.”*

Similarly, FS-6 voiced: *“Being a female at university means proving that I can handle both my academic work and all the roles in society that I play at home in my family and socially. It is a constant battle*,* and it is exhausting.”*

These explanations emphasize the gendered dimension of academic stress where female student often face the dual challenge of excelling academically while also adhering to social expectations regarding their roles at home and in society. This is a double burden that leads to heightened stress levels and creates a situation where female students feel uncertain between meeting their academic demands and their personal and family obligations.

### Physical and mental toll

The physical and mental toll of academic stress was evident across the interviews. Many female students reported that the constant pressure to perform academically, combined with social expectations, manifested in physical and mental health issues. Commonly reported symptoms included fatigue, difficulty concentrating, anxiety, and sleep disturbances. As FS4 said:

*“I have had days where I am just mentally drained. I cannot focus anymore*,* and I end up with a headache. I also struggle to sleep at night*,* thinking about the workload and the pressure of everything that needs to get done.”*

Another FS6 stated that: “*The stress is not just mental*,* it is physical. I feel tired all the time*,* which makes it harder to concentrate*,* and I often feel physically exhausted by the end of the day. I feel like my body is constantly in a state of alertness because of the academic stress.”*

This highlights that the significant toll academic stress takes on female students extends beyond academic performance to their overall health and well-being. Taken together, these accounts show that participants experienced academic stress as cumulative and embodied rather than as a response to isolated academic events. Persistent demands affected concentration, sleep, energy, and their capacity to recover from academic pressure.

### Psychological capital and coping strategies

#### Resilience and persistence

Resilience emerged as a key theme in how female students managed academic stress. The ability to persist through challenges and setbacks was frequently mentioned by participants as an essential quality in dealing with academic pressures. Female student described resilience as an internal strength, built over time through their experiences of overcoming difficulties, which helps them stay focused and motivated during tough academic periods. Many participants highlighted that resilience was not just about surviving stress but about actively learning and growing from it. The more challenges they faced, the stronger and more persistent they felt as FS3 shared:

*"When stress becomes overwhelming*,* I just remind myself that I have managed challenging situations before and I can do it again. I think resilience is about finding a way to keep moving forward even when it feels impossible.”*

This reflection shows how past experiences of overcoming stress become a resource for future challenges, reinforcing the student’s ability to cope with current pressure. Similarly, FS6 stated that:

*“The pressure gets to me*,* but my ability to push through and keep going is something I have developed over time. Every time I overcome a challenge; it just makes me more resilient for the next one.”*

The connection between these two female experiences shows how resilience is not only about surviving stress but also growing through it. Both students emphasize the role of previous experiences in building strength for future challenges. Overcoming stress makes them more confident and persistent in dealing with new problems.

#### Optimism and hope

Optimism and hope were frequently identified as significant motivators for female students dealing with academic stress. Female students who maintained a positive outlook on their academic journey reported being better able to manage stress and stay motivated even during particularly difficult periods. The belief that things would improve, even when the immediate situation seemed overwhelming, helped sustain their efforts and kept them engaged with their academic goals, as FS7 shared.

*“I tried to keep a positive attitude even when things seemed tough*,* I remind myself that this stress is temporary. Hope that things will improve*,* keep me motivated to continue working hard.”*

In a similar vein, FS4 said:

*“For me*,* hope is a powerful thing*,* it is what keeps me moving forward when everything else feels like it is too much.”*

Together, these reflections highlighted the critical role of hope as a sustaining force. For both FS7 and FS4, hope serves as a shield against overwhelming stress, enabling them to persevere through academic challenges, knowing that the current stress is only temporary and that things will improve.

#### Self-efficacy and confidence

Self-efficacy, or the belief in one’s ability to succeed, played a key role in how female students coped with academic stress. Students who felt confident in their abilities were more likely to face stress with a sense of control, believing their efforts would lead to success. Self-efficacy was a key factor that helped students approach challenges confidently, reducing feelings of helplessness or anxiety. Female students with high self-efficacy felt capable of handling difficult situations, which made it easier for them to manage stress effectively, as FS10 explained:

*“I have always believed that if I put in the effort*,* I can succeed. That confidence in my abilities makes it easier to handle the stress because I know I can get through it.”*

This belief in personal ability helps manage stress by providing a sense of control over the situation. In the same light, FS2 reflected.

*“When I have faith in my abilities*,* I feel less stressed because I know I can do it. Self-efficacy gives me the courage to keep moving even when the pressure is powerful.”*

Both students emphasize how self-efficacy acts as a confidence booster, reducing the emotional toll of stress. By maintaining confidence in their capabilities, they can stay calm under pressure and tackle challenges with a sense of control, making stress more manageable. Across the psychological capital subthemes, resilience, hope, optimism, and self-efficacy functioned as connected coping resources rather than isolated qualities. Previous experiences of overcoming difficulties strengthened confidence and persistence, while hope and optimism supported continued engagement with future academic goals.

### Role of social support

#### Emotional and practical support from family and friends

Social support from family and friends was identified as a critical resource for coping with academic stress. Many participants described how their families provided emotional reassurance, offering reminders to take breaks and maintain balance. In addition, friends were a key source of academic support, forming study groups and sharing resources to lighten the workload. This type of support helped reduce feelings of isolation and stress, providing a sense of community that made academic challenges more manageable, as FS 13 mentioned.

*“Whenever I feel overwhelmed*,* my family member reminds me to stay calm and keep my perspective. My friends also help a lot; they study with me*,* and that makes the workload feel lighter.”*

Similarly, another female student highlights the importance of family and friends in alleviating academic stress, as FS1 voiced:

*“I share my worries with my close friend*,* which really helps. We exchange study tips and help each other out when things get tough.”*

These voices highlight the importance of both family and friends in reducing the emotional burden of academic stress. F13 views family as an emotional anchor, emphasizing how this grounding presence helps provide stability. Similarly, the FS1 statement highlights the collaborative nature of peer support, in which study tips and mutual encouragement lighten the academic workload and create a shared experience that reduces stress.

#### Peers and mentor support

In addition to family and friends, many participants emphasized support from peers and mentors, who provided not only academic guidance but also emotional support, helping students navigate the complexities of balancing academic work with personal life. Peer support on the other hand, was crucial in creating a sense of solidarity where students could share their experiences, discuss stressors, and encourage one another. These relationships allowed students to feel less isolated in their academic struggles and more empowered to overcome challenges as FS11 shared her experience.

*“My mentor has helped me develop time management skills and stay on track. She has been invaluable in guiding me through the stress of balancing academic and personal life.”*

On the other hand, FS15 stated:

*“Studying with my peer is so important*,* we get through the stress together*,* and we can always talk about what is stressing us out and offer support.”*

Both responses highlight the dual role of mentors and peers in reducing academic stress. FS11 emphasizes how mentors not only offer academic guidance but also help students develop essential skills, such as time management, to cope with university stress. FS15, on the other hand, underscores the collective nature of peer support, where mutual encouragement and collaboration help students share the burden of academic stress.

#### Roles of social networks in stress management

Social networks, including family, friends, and mentors, played a significant role in managing academic stress. The emotional reassurance, guidance, and practical support from these networks helped to create a buffer against the pressures of university life. Participants frequently highlighted how these social connections made it easier to manage stress by providing a sense of belonging, perspective, and shared responsibilities for success. Whether it was through family support at home or peer collaborations in study groups, these networks enabled students to feel less isolated in their struggles, as FS 10 noted:

*“I cannot imagine going through university without my support network. Whether it is my family*,* reassuring my friends studying with me*,* or me.”*

It reflects on how the presence of family and friends during stressful times offers not only reassurance but also practical help, such as studying together, which eases the burden of university life. Building on this, FS2 highlights the significance of familial support in overcoming academic stress. FS2 shared:

*“Whenever I feel overwhelmed*,* my family reminds me to take a step back and priorities self-care. It helps to know that I am not alone in this and that someone got me back.”*

It is revealed that interpersonal relationships, especially with family and friends, play a pivotal role in helping students cope with academic stress. These support systems provide not only emotional reassurance but also practical strategies, such as prioritizing self-care and staying together, which empower students to navigate the pressures of university life with resilience and confidence. Across these accounts, social support operated through three main processes: emotional reassurance reduced feelings of isolation, practical collaboration helped participants manage academic demands, and guidance from peers and mentors supported problem solving and time management. However, these relational contexts were not uniformly protective. When considered together with the gendered experiences reported below, family and social relationships could provide support while also carrying expectations and responsibilities that increased participants’ coping demands.

### Gendered barriers shaping coping with academic stress

Female students’ experience of academic stress is deeply influenced by gendered expectations and societal roles. This theme captures how gendered conditions shaped participants’ capacity to cope, rather than simply describing another source of academic stress. They not only faced the typical academic pressures of coursework and deadlines but also had to negotiate societal and cultural norms that shape their academic experiences. These gendered expectations often exacerbated their stress and contributed to a unique set of challenges in balancing academic responsibilities with traditional gender roles.

Female students highlighted that those societal expectations to excel academically while also fulfilling traditional roles as caregivers added extra stress. Many female students mentioned how their academic performance was judged in relation to their family duties, increasing both their workload and emotional strain, as FS6 explained:

*“At home*,* I must help with chores and take care of my younger siblings*,* but at the same time*,* I need to maintain high academic performance. It is like balancing two full-time jobs*,* and it is exhausting.”*

On the other hand, FS3 shared her experience, and she said:

*“There is this feeling that I cannot afford to fail*,* not just academically but also as a daughter and sister. Society expects women to do it all*,* and that adds to the pressure.”*

Both female students’ experiences highlight the compounded stress they face. Societal expectations to balance family responsibilities with academic demands create a burden, making it difficult to focus solely on their academic work. Importantly, these responsibilities also shaped coping by reducing the time and attention available for studying, rest, and self-care. Gendered family expectations therefore operated not only as additional stressors but also as constraints on the resources participants could devote to managing academic pressure.

#### Gender bias and discrimination in academic settings

Gender bias and discrimination in academic settings emerged as a significant stressor for female students. Many participants noted that their academic ideas and contributions were often overlooked, particularly in male-dominated academic spaces or group settings. Female students felt they had to work harder to be taken seriously and recognized for their academic abilities, as FS9 noted.

*“I have often felt that my contributions in class are not taken as seriously as my male peers. Sometimes it feels like I must work twice as hard just to be hard.”*

Similarly, another FS 7 shared her experience:

*“In group projects*,* my ideas are often dismissed. It feels like I am constantly battling to prove myself academically.”*

These experiences of gender bias reflect the wider issue of female underrepresentation in academic leadership and decision-making roles. Such bases not only affect the recognition of female students’ academic work but also diminish their academic agency, which can further contribute to stress and feelings of exclusion. For these participants, coping therefore involved not only managing academic demands but also expending additional effort to establish their academic credibility in settings where they perceived their contributions as being taken less seriously.

#### Balancing academic and social roles

Female students frequently spoke about the emotional labor they were expected to perform in both academic and social contexts. Many felt an added responsibility to maintain social harmony, provide emotional support to peer, and fulfill nurturing roles in their personal lives. This additional emotional labor, which is often invisible and undervalued, is compounded by their academic stress. As FS4 voiced:

*“It is not just about studying hard*,* I am also expected to be the emotional support for my friends*,* manage family expectations and stay involved in social activities. It is likely being expected to be everything to everyone.”*

FS2 further elaborates on this by sharing how the constant pressure to be the perfect student and emotionally available to others often leaves her feeling drained with time for self-care, as she said:

*“There is a constant pressure to be the perfect student and emotionally available to others. This emotional burden often drains me and leaves little room for self-care or relaxation.”*

These reflections highlight the extra emotional labor that female students are often expected to undertake, which further exacerbates their stress levels and makes it harder for them to focus solely on their academic goals. Here, gendered coping constraints were evident in the competition between caring for others and maintaining participants’ own opportunities for recovery and self-care.

Taken together, Themes 3 and 4 directly address RQ3. Social support helped participants cope through reassurance, practical assistance, shared academic work, guidance, and a sense of not facing academic pressure alone. At the same time, gendered barriers shaped these coping processes by adding domestic and emotional responsibilities, limiting time for rest and self-care, and requiring additional effort in response to perceived gender bias. The findings therefore show that participants’ social environments could function as sources of support while gendered expectations within family, social, and academic contexts could simultaneously increase the demands they had to manage.

## Discussion

This study explores academic stress among female students at a Chinese university through a qualitative approach, analyzing the perceptions of 15 female students. This study highlights the role of psychological capital and social support in coping with academic stress. The findings indicate that psychological capital and social support helped participants cope with academic stress, whereas gendered family and social expectations intensified the pressures they experienced.

Psychological capital, including resilience, optimism, and self-efficacy, was found to play a crucial role in helping female students manage academic stress. Resilience, in particular, was identified as essential for navigating academic challenges. These findings align with research by Rasheed and Bhat [31], which emphasize the importance of psychological capital in enhancing an individual’s ability to cope with adversity. Female students in this study often relied on their inner strength to persist despite academic pressures. However, they were also highly dependent on external support systems to manage stress effectively. Optimism and self-efficacy were also important in maintaining academic motivation. Female students reported that their belief in their abilities helped them stay positive despite overwhelming academic pressure. These results are consistent with Rasheed and Sultan [32], found that self-efficacy influences an individual’s ability to succeed in challenging situations.

The study identified social support from family, friends, and mentors as an important factor in managing academic stress. Similar to studies by Ullah, Akhter [22], and [33] found that emotional support and practical help from a close social network were key in helping female students manage challenges and reduce academic stress. Furthermore, our study explores that female students are often expected to balance academic responsibilities with family obligations, contributing to a double burden that increases their stress level. The findings of this study resonate with Ullah, Akhter [22], has highlighted how the gendered division of labor within families often places additional expectations on female students. These societal pressures compounded the participants’ academic stress, leading to feelings of being overwhelmed and unable to fully focus on their academic work. Moreover, participant experiences of gender bias and discrimination within academic settings were consistent with previous studies, such as Song and Ji [34], reported that female students often face underrepresentation in leadership roles and gendered expectations in academic environments. Many of the participants in our study noted that their ideas were often overlooked or dismissed in group settings, which further exacerbated their stress levels and diminished their sense of academic agency.

The reported experiences of gendered expectations, competing responsibilities, and perceived gender bias also have potential implications for institutional support. Although insufficient gender-sensitive institutional support did not emerge as a distinct theme in the present analysis, the findings suggest that universities may consider support approaches that are responsive to the academic and gender-related pressures described by participants. Gender-sensitive mental health support and academic mentoring may be relevant areas for consideration, although these specific interventions were not evaluated in this study. Oganetse and Phiri [35], observed that while universities may offer general academic support, gender-sensitive programs are often inadequate. Additionally, Hagan [36] advocates programs that build self-efficacy and leadership skills among female students to better equip them to manage academic stress and overcome gendered barriers in higher education. Participants’ reports of having their academic contributions overlooked or dismissed also suggest a broader need for universities to consider how gender bias may operate within academic environments. These institutional considerations should be understood as potential implications of the present findings rather than as interventions or deficiencies directly established by this study. Naylor and Mifsud [37], argued that addressing structural inequities is important for fostering an inclusive and equitable academic environment.

### Limitations and transferability

The findings should be interpreted in light of the study’s sampling characteristics. All participants were recruited from a single Chinese university and were drawn from three related social science disciplines, education, psychology, and sociology. Although this relatively focused sample enabled an in-depth exploration of shared experiences within these academic contexts, students from other disciplines, institutions, or regions of China may experience academic stress, gendered expectations, psychological resources, and social support differently. The relatively small purposive sample of 15 participants may also have introduced self-selection, as students who were willing to discuss their experiences of academic stress may differ from those who did not participate. In addition, eligibility was based on participants’ self-reported experience of moderate to high academic stress rather than assessment using a standardized measure.

The study included only female students and did not include male participants or another comparison group. Therefore, the findings cannot determine whether the experiences identified are specific to female students or are also experienced by other student groups. Interviews were conducted in both Chinese and English, and Chinese-language interview material was translated into English by the second author and reviewed by the research team for accuracy. Despite this verification process, some culturally or linguistically specific meanings may have been altered or lost during translation.

Researcher characteristics and positionality may also have influenced data collection and interpretation. In particular, the first author was a male Pakistani international researcher interviewing female Chinese students about potentially sensitive gendered experiences, which may have influenced how some participants described or disclosed their experiences. The research team’s different gender, cultural, linguistic, and institutional positions may also have shaped interpretation and theme development. These influences were considered during team discussion and refinement of the themes.

Therefore, the findings are not intended to represent all female university students in China. Instead, their transferability should be considered in relation to settings with similar institutional, disciplinary, and sociocultural characteristics. Future qualitative research could include female students from a wider range of disciplines and universities to examine whether similar themes emerge across different higher education contexts. Including more diverse institutional settings, regions, disciplines, and comparative participant groups may also help determine which findings are shared across contexts and which are context-specific.

### Conclusion and recommendations

This study highlights the challenges faced by female students in managing academic stress at a Chinese university. Psychological capital, including resilience, hope, optimism, and self-efficacy, helped participants manage academic pressure and maintain motivation. Social support from family, friends, peers, and mentors also provided emotional and practical assistance. However, participants’ accounts showed that gendered family and social expectations could simultaneously increase stress by adding caregiving, domestic, and emotional responsibilities. Female students are often pressured to excel academically while also fulfilling traditional gender roles, and participants described associated fatigue, anxiety, sleep difficulties, reduced time for self-care, and difficulty focusing on their academic work.

As potential implications of these findings, universities may consider strengthening accessible mental health support, mentoring, and peer-support initiatives that are sensitive to gendered academic and social pressures. Universities may also consider efforts to address gender bias within academic settings and to support female students in managing competing academic and personal demands. These recommendations are broader implications derived from participants’ experiences and were not directly evaluated in this study. Further research across different institutions and disciplines is needed to identify which institutional interventions are most appropriate and effective.

## Data Availability

No datasets were generated or analysed during the current study.
